# Nanoparticle Superlattices: The Roles of Soft Ligands

**DOI:** 10.1002/advs.201700179

**Published:** 2017-09-06

**Authors:** Kae Jye Si, Yi Chen, Qianqian Shi, Wenlong Cheng

**Affiliations:** ^1^ Department of Chemical Engineering Faculty of Engineering Monash University Clayton 3800 Victoria Australia; ^2^ The Melbourne Centre for Nanofabrication 151 Wellington Road Clayton 3168 Victoria Australia; ^3^ State Key Laboratory of Bioelectronics Jiangsu Key Laboratory for Biomaterials and Devices School of Biological Science and Medical Engineering Southeast University Nanjing China

**Keywords:** DNA, molecules, nanoparticles, polymers, soft ligands, superlattices

## Abstract

Nanoparticle superlattices are periodic arrays of nanoscale inorganic building blocks including metal nanoparticles, quantum dots and magnetic nanoparticles. Such assemblies can exhibit exciting new collective properties different from those of individual nanoparticle or corresponding bulk materials. However, fabrication of nanoparticle superlattices is nontrivial because nanoparticles are notoriously difficult to manipulate due to complex nanoscale forces among them. An effective way to manipulate these nanoscale forces is to use soft ligands, which can prevent nanoparticles from disordered aggregation, fine‐tune the interparticle potential as well as program lattice structures and interparticle distances – the two key parameters governing superlattice properties. This article aims to review the up‐to‐date advances of superlattices from the viewpoint of soft ligands. We first describe the theories and design principles of soft‐ligand‐based approach and then thoroughly cover experimental techniques developed from soft ligands such as molecules, polymer and DNA. Finally, we discuss the remaining challenges and future perspectives in nanoparticle superlattices.

## 1 Introduction

Nanoparticles were coined as “artificial atoms” due to their unique properties originating from their nanoscale dimensions.^1^ To date, wet chemistry has demonstrated to be a powerful scalable route to synthesize nanoparticles with well‐defined sizes and shapes, enabling the formation of “artificial periodic table”.^2^ The full potential of the elements in the table cannot be realized until one is able to manipulate nanoparticles at will into well‐defined assemblies, which is critical for their downstream applications in future optoelectronic devices, biological diagnostics and energy‐harvesting systems.

Nanoparticle superlattices are one kind of such well‐defined assemblies, which are a new class of crystalline materials with collective properties different from those of bulk phase crystals, isolated nanocrystals and even disordered nanocrystal assemblies.^3^, ^4^, ^5^, ^6^, ^7^, ^8^, ^9^, ^10^ For instance,

- Coherent vibrational modes can only appear in highly ordered nanoparticle superlattices,^3^ and synergistic effects in superlattices can lead to enhanced p‐type conductivity;^5^
- 2D DNA‐metal nanoparticle sheets exhibited unusual mechanical properties;^11^
- 2D polymer‐nanorod superlattice sheets exhibited novel plasmonic properties dependent on packing order.^12^

The concept of nanoparticle superlattices was first introduced by Janos Fendler in 1994,^13^, ^14^ and in the subsequent year, CB Murray reported the first three dimensional quantum dot nanoparticle superlattice.^15^ This paper catalyzed a series of developments by a number of research groups. Unusual properties including spin properties,^16^ metal‐insulator transition,^17^ mechanical properties,^18^ p‐type conductivities,^5^ vibrational coherence,^3^ electronic properties^19^ and plasmonic properties^11^, ^12^, ^20^ have all been found and investigated. In addition, diverse types of lattice structures have been found including face‐centered cubic (FCC), body‐centered cubic (BCC), hexagonal‐closed packed (HCP), diamond‐like lattice, and lattice structures not existing in nature.^21^, ^22^

Construction of nanoparticle superlattices can be efficiently achieved by bottom‐up self‐assembly strategy in an equilibrium or non‐quilibrium process. Either process involves rational design of particle‐capping organic species (termed soft ligands) in order to achieve high‐quality ordered assemblies. In the equilibrium self‐assembly, well‐defined ethalpic ligands are usually used to functionalize nanoparticles so that particle‐to‐particle ethalpic binding interactions in a fluids can be controlled and programmed. An elegant example is the DNA‐based nanoparticle superlattice system in which the binding forces can be controlled by the Watson‐Crick base‐pairing interactions under optimum annealing temerpatures. The ordered nanoparticle assemblies can also be achieved in an entropy‐driven self‐assembly strategy, such as by the widely‐used drying‐mediated process. Drying is usually a stochastic process, leading to frequently observed kinetically trapped non‐equilibrium structures. It is possible to control spatial and temporal drying events using micropatterned surfaces or other substrates to achieved well‐ordered nanoparticle superlattices. In addition to the fabrication, the soft ligands also play critical roles in regulation of the lattice structures including nearest‐neighbor spacing and symetry as well as superlattice properties.

**Figure 1:**
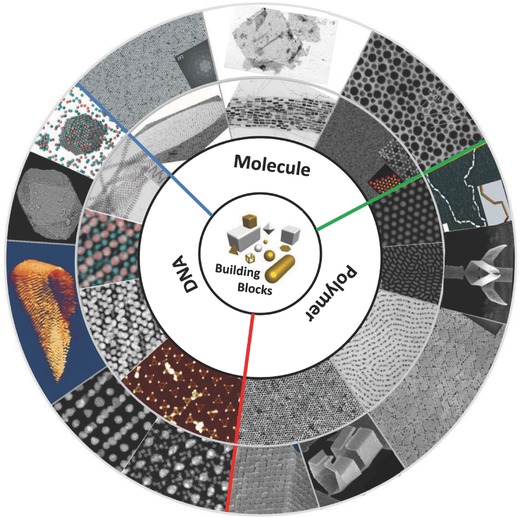
Schematic summary of various nanoparticle superlattices assembled from libraries of soft‐ligand capped nanocrystals using molecule‐based, DNA‐based and polymer‐based assemblies. Reproduced with permission.^10^, ^11^, ^12^, ^18^, ^20^, ^21^, ^79^, ^120^, ^133^, ^135^, ^138^, ^144^, ^171^, ^172^, ^173^, ^174^, ^175^, ^176^, ^177^ Copyright 1998, 2001, 2006, 2007, 2009, 2010, 2011, 2013, Nature Publishing Group. Copyright 2008, 2011, 2014, American Chemical Society. Copyright 2010, 2013, John Wiley and Sons. Copyright 2006, The American Association for the Advancement of Science.

To date, a few reviews have discussed nanoparticle superlattices,^1^, ^2^, ^23^, ^24^, ^25^, ^26^, ^27^, ^28^, ^29^, ^30^, ^31^, ^32^, ^33^, ^34^ however, a dedicated review emphasizing the important roles of soft ligands has not yet reported to the best of our knowledge. We therefore are motivated to cover the recent advances in nanoparticle superlattices from viewpoint of soft ligands. Particularly, we mainly focus on superlattices from soft corona/solid core nanoparticles in which linear molecular, polymeric or DNA ligands cap uniformly solid inorganic nanoparticle surfaces. Such soft corona/solid core nanoparticles could lead to a variety of one‐, two‐ and three‐dimensional superlattices (**Figure** **1**). The typical roles of soft ligands in manipulating self‐assembly of inorganic nanoparticles include: a) to prevent inorganic nanoparticles from disordered aggregation by introducing steric hindrance which counteract with dominant van der Waals and electrostatic forces; b) to adjust the interparticle potential by regulating nanoscale forces; c) to program lattice structures such as by DNA molecules; d) to maintain mechanical superlattice integrity as a whole via strong ligand‐ligand interactions.

Here, we will discuss how soft ligands including molecules, polymer and DNA, have been used in directing the formation of nanoparticle superlattices. We describe both theoretical and experimental aspects of soft ligand‐based assembly of superlattices. Finally, we discuss the remaining challenges and future perspectives in nanoparticle superlattices.

## 2 General Design Principles of Soft Ligand‐Based Nanoparticle Superlattices

The interactions between inorganic nanoparticles are quite complex, involving various forces at different temporal and spatial scales, including van der Waals forces, electrostatic forces, dipole‐dipole interactions, etc.^23^ It has been demonstrated that soft ligands could be used to manipulate these complex forces to direct the assembly of nanoparticles into superlattices. Depending on the types of soft ligands, various theoretical models have been proposed to interpret the self‐assembly of ligand‐capped nanoparticles. In this section, we will discuss on the three main thermodynamic models that can be used as a guideline for superlattice design – (1) soft sphere model, (2) entropic spring model and (3) complementary contact model. The first two theory models apply for elucidating the behavior of soft organic ligands in entropy‐driven nanoparticle assembly systems, whereas the latter applies for enthalpically‐favorable assembly systems, in particular for DNA‐functionalized nanoparticles.

### 2.1 Soft Sphere Model

Soft ligand capped nanoparticles behave more like ‘soft spheres’ during self‐assembly of superlattices.^35^ Unlike hard spheres,^36^, ^37^ the interaction potential in soft spheres is actually contributed from the van der Waals (vdW) attractive forces between nanoparticle hard cores, and steric repulsion of soft ligands.^38^ Korgel et al. proposed the soft sphere model,^35^ in which the interaction potential (*U_total_*) can be quantitatively estimated as the combined contributions from vdW attraction potential (*U_vdW_*) and steric repulsion potential (*U_steric_*),

(1) $$U_{total}=U_{vdW}+U_{steric}$$

Using Hamaker's derivation,^39^ the van der Waals interaction can be estimated between a pair of nanospheres with diameter (*d_core_*) and nearest neighbor core‐to‐core distance (*d_NN_*),

(2) $$U_{vdW}=-\frac{A}{12}\left[\frac{d_{core}^{2}}{d_{NN}^{2}-d_{core}^{2}}+\frac{d_{core}^{2}}{d_{NN}^{2}}+2\ln\left(\frac{d_{NN}^{2}-d_{core}^{2}}{d_{NN}^{2}}\right)\right]$$

where *A* is the Hamaker constant that depends on the polarizability of nanoparticle and surrounding medium. The steric repulsion depends on both ligand‐solvent and ligand‐ligand interactions.^40^ In a good solvent, *U_steric_* can be calculated by employing de Gennes' expression,^41^

(3) $$U_{steric}=\frac{100Rh_{0}^{2}}{\left(d_{NN}-d_{core}\right)\pi \sigma^{3}}k_{B}T\exp\left[-\pi \left(d_{NN}-d_{core}\right)/h_{0}\right]$$

where *h_0_* is the ligand brush thickness and σ is the diameter of the circular footprint occupied by a single ligand on the nanoparticle surface. The total interaction potential is a balance between van der Waals and steric hindrance potentials, depending on the various parameters in Equations (2) and (3), including the diameter and composition of nanoparticle, as well as the length and conformation of soft ligands. In addition, other types of forces can also influence superlattice formation, such as electrostatic attraction/repulsion between surface‐charged particles,^42^ permanent magnetic moments between semiconducting nanoparticles^10^, ^43^ and Watson‐Crick base‐pairing interaction from DNA‐capped nanoparticles.^2^, ^44^, ^45^, ^46^, ^47^ It is important to have these forces considered carefully when designing nanoparticle superlattices.

### 2.2 Entropic Spring Model

**Figure 2:**
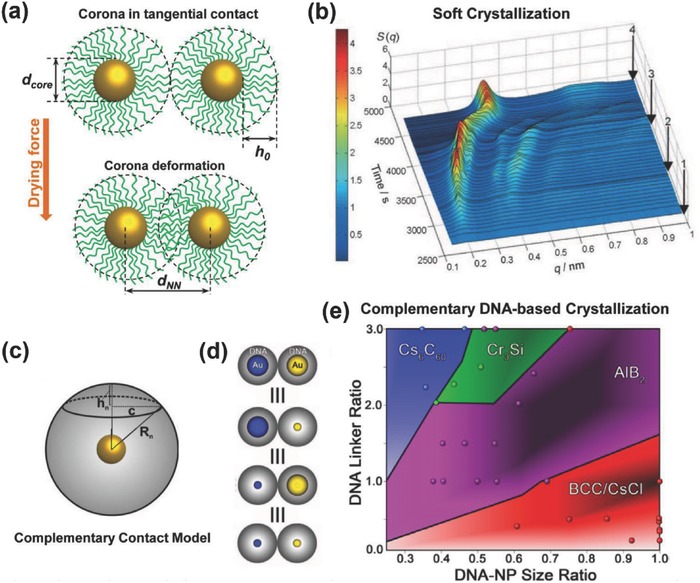
Design principles to soft‐ligand based nanoparticle superlattices. a) Scheme for the deformation of soft‐corona nanoparticles in soft‐crystallization. b) Probing of soft crystallization using in situ SAXS demonstrates the continuous deformation of corona. Reproduced with permission.^48^ c) Scheme of DNA‐capped hydrodynamic nanoparticle in complementary contact model. The spherical cap represent overlapped portion of corona with adjacent nanoparticle. d) Illustration of design rule (3) that nanoparticles with same hydrodynamic size form similar superlattice structure. e) Phase diagram shows predictable superlattice structure by controlling core size and DNA ligand ratio. Each coloured domain denotes the specific superlattice structure. The dots represent the superlattice obtain in experimental. Reproduced with permission.^7^ Copyright 2011, Nature Publishing Group.

The soft ligand‐based superlattices usually formed in a drying‐mediated self‐assembly process, in which Cheng et al. proposed the entropic spring model.^48^ In a drying‐mediated self‐assembly of organically‐capped nanoparticles, their organic corona must deform dynamically during dewetting process due to surface tensions. Using single stranded DNA (ssDNA) capped gold nanoparticles as a model system (**Figure** **2**a), two dimensionless groups, the softness, χ and deformation parameter, λ were defined,

(4) $$\chi =2h_{0}/d_{core}$$

(5) $$\lambda =\frac{2h_{0}-\left(d_{NN}-d_{core}\right)}{2h_{0}+d_{core}}$$

where *d_core_* is the diameter of nanospheres and *d_NN_* is nearest neighbor core‐to‐core distance of nanospheres. With this definition, the typical softness for ssDNA‐capped gold nanoparticles is between 0.6 and 5.1, which is a much wider range than that for alkyl‐capped nanoparticles (0.3–0.8). For the typical polystyrene‐capped gold nanoparticles reported recently,^49^ the softness value can be achieved with a value between 1.6 and 2.5 using commercially available thiolated polystyrenes.

DNA molecules can form a brush like layer on highly curved nanoparticle surfaces – a situation similar to 2D solid surface but follow a different scaling law. Based on the two dimensionless groups defined, an entropic spring model could be derived by assuming organic capping ligands as molecular springs and applying brush polymer theory,^50^

(6) $$\lambda =\frac{2fb^{4/3}}{3k_{B}T{\left(\gamma \sigma \right)}^{1/3}}\frac{\chi }{1+\chi }$$

where *b* is the Kuhn length, *k_B_* is the Boltzmann constant, *T* is temperature, γ is scaling constant (including excluded volume effects), *f* is the applied force and σ is the grafting density of ligand. This equation predicts that the deformation parameter is solely dependent on the particle softness when the applied force is constant and solely dependent on the applied force when the particle softness is fixed.

This model was proved by using Synchrotron‐based small‐angle X‐ray scattering (SAXS) which enabled *real‐time* monitoring dynamic crystallization process of ssDNA‐capped gold nanoparticles (Figure 2b). The full picture of temporal process was revealed by monitoring the entire self‐assembly process. The softness, χ, could be estimated from dynamic light scattering (giving overall dimensions) and transmission electron microscopy (giving core size). The deformation parameter, λ, could be estimated from the value of nearest neighboring spacing, *d_NN_* which could be calculated from Synchrotron‐based SAXS pattern. It appears that the model is generic, applicable to alkyl‐capped gold nanoparticles and stretched DNA‐nanoparticle superlattices, and anisotropic nanowire assemblies.^51^, ^52^

### 2.3 Complementary Contact Model

The above two theory models can be used to explain well the thermodynamics and kinetics of drying‐mediated self‐assembly of organically‐capped inorganic nanoparticles. Departing from this, DNA can serve as a unique programmable ligand due to their unique Watson‐Crick base‐pairing interactions, leading to a number of interesting superlattice structures.^7^, ^53^ In general, multiple forces exist between DNA‐capped nanoparticles, including Watson‐Crick base pairing forces, pi‐pi stacking interactions, van der Waals (vdWs) attractions, and electrostatic repulsive forces. The latter two often play a negligible role, especially when long DNA ligand is used. Hence, under aqueous buffered environments, DNA base‐pairing forces play a critical role in superlattice growth, which could be controlled by ionic strength and/or annealing temperature.^45^

A complementary contact model (CCM) was proposed for DNA‐based nanoparticle superlattice growth in aqueous buffered conditions.^7^ In this model, complementary DNA sticky ends are assumed to be able to physically contact with each other, and any contacted sticky ends will eventually form a DNA duplex. Also, DNA‐nanoparticles are treated as ‘fuzzy spheres’ where the sticky ends are confined in a predictable region surrounding the nanoparticles (Figure 2c). The maximum radius (*R_n_*) of DNA‐nanoparticle in superlattice can then be calculated as:

(7) $$R_{n}=r_{n}+0.34\cdot x+0.4$$

where *r_n_* is the radius of the nanoparticle *n*, and *x* is the number of DNA bases. If the overlapped portion of DNA corona with neighbor nanoparticles is considered as spherical caps, then the surface area (*S_n_*) of adjacent fuzzy spheres is:

(8) $$S_{n}=\pi \left(c^{2}+h_{n}^{2}\right)$$

where *c* and *h_n_* represent the radius and height of the overlapped spherical cap. Then the number (*N_duplex,n_*) of hybridized DNA strands on nanoparticle *n* can be calculated as:

(9) $$N_{duplex,n}=S_{n}N_{neighbor,n}N_{DNA,n}N_{particle,n}/4\pi R_{n}^{2}$$

where *N_neighbor,n_*, *N_DNA,n_*, *N_particle,n_* represent the number of neighboring particles, DNA capped to particle *n*, and *n*‐type nanoparticles for a unit cell, respectively. By applying this model, a phase diagram has been simulated for predicting crystallographic structures as a function of nanoparticle size ratio and DNA ligand ratio (Figure 2d and e), which is demonstrated to be consistent with the experimental results. The basic qualitative design principles have been outlined for DNA‐based superlattices:^7^, ^53^ (1) DNA‐NPs (with equal *R_n_*) will maximize the number of closest neighbors with which it can be hybridized; (2) slowing down the DNA linker releasing and rehybridization rate would facilitate lattice growth and stabilize kinetic products; (3) the hydrodynamic size, instead of core size or DNA length, mainly determines the superlattice structure; (4) for binary components, the stabilized superlattice is mainly determined by the hydrodynamic size ratio and DNA ligand ratio between two building blocks; (5) a nanoparticle can be capped with different linkers that contain different base sequences, which can lead to complex superlattices.

## 3 Molecule Mediated Nanoparticle Superlattice

The first molecule‐based nanoparticle superlattices were reported by Janos Fendler, in which oleic acid‐protected silver nanoparticles^14^ and dodecylbenzenesulfonic acid‐protected cadmium sulfide nanoparticles^13^ were assembled at air‐water interface using Langmuir‐Blodgett technique. Despite only small‐scaled ordering achieved, this seminar work simulated the early development of molecule‐mediated nanoparticle superlattices.^15^, ^35^, ^54^, ^55^ Typically, molecular ligands serve as stabilizers to prevent nanoparticle aggregation, and contribute to superlattice formation via ligand interdigitation.^56^, ^57^ The ligand‐nanoparticle and ligand‐ligand interactions are the key factors determine the superlattice formation: for the former, end‐functionalized molecular ligands are often used to form self‐assembled monolayers (SAMs) on nanoparticle surfaces; the later will determine interparticle potentials by using hydrophobic, electrostatic, hydrogen bonding interactions between ligands, etc. **Table** **1** shows the different kinds of molecular ligands and their headgroups which bind specifically to certain metallic nanoparticles or quantum dots. The binding interactions between the ligand and nanoparticle surface reflected their ability to successfully assemble into superlattices.^58^ Depending on different types of materials, specific headgroups have been successfully used. As for gold nanoparticles, thiols are generally able to form better superlattices due to their strong binding to particle surface, enabling dense standing packing of alkyl chains to form SAMs with an all‐trans conformation.^59^ The weaker surface binding often leads to low number density and disorder of the exterior alkyl chains.

**Table 1:** Summary of the different kinds of ligands that have been used for molecule‐based nanoparticle superlattice

Functional headgroup which binds to nanoparticle	Ligand used	Nanoparticle type	Nanoparticle assemblies	Reference
—COOH	Oleic acid	CoO	2D assemblies	56
		Fe_2_O_3_	2D hcp superlattices	205
			Binary superlattices	42, 97
		Fe_3_O_4_	1D superlattice chains	102
			2D and 3D supraparticles	104, 206
		PbS	Binary superlattices	42, 97
			3D fcc superlattices	207
			bct superlattice and bilayered hesagonal lattice	86
		Ag particles	2D lattices	14
		Ni particles	3D fcc/hcp superlattices	146
		Pt particles	3D fcc superlattices	87
		PbSe	2D honeycomb superlattice	208
		CeO_2_	fcc/hcp/hnc/cubic superlattices	103
	Thioglycolic acid	CdTe	1D crystalline nanowires	209
			Twisted ribbons	72
	Stearic acid	Fe_3_O_4_	1D and 2D assemblies	210
		Fe_2_O_3_		
	N‐acetylglutathione	Au	3D superlattice	77
	Dodecylbenzenesulfonic acid	CdS	Monoparticulate layers	13
—NH_2_	Oleyamine	Fe/Fe_3_O4	2D assemblies	56
		Au wires	2D binary superlattice film	198
	Octadecylamine	Co	Hcp disk shaped superlattices	211
	Trioctylamine			
	Tributylamine			
	Dodecylamine	Au spheres	2D & 3D superlattices	58, 70
—P	Trioctylphosphine	Cds	Semiconductor nanocrystallites	212
		CdSe	Semiconductor nanocrystallites	212
		CdTe	Semiconductor nanocrystallites	212
		Au sphere	2D & 3D superlattices	58
—P=O	Trioctylphosphine oxide	Co	Hcp disk shaped superlattices	211
		CdSe	Hexagonal superlattice	213
		PbS	Hexagonal 2D superlattices	214
			Binary superlattices	42
—SH	Propanethiol	Ag octahedra	2D superlattices	105
	Mercaptosuccinic acid	Au sphere	2D superlattices	79
			3D superlattices	79, 215
	Dodecanethiol	Au sphere	2D superlattices	18, 55, 56, 58, 73, 82, 92, 216
			2D binary superlattice films	42, 97, 198, 216
			3D superlattices	82, 217, 218
		Au rod	2D binary superlattice films	198
		Ag QDs	2D superlattices	17
		Ag_2_S	2D superlattices	219
		Ag particles	2D superlattices	35
			3D superlattices	93, 219
			f.c.c multilayer superlattices	220
			2D binary superlattices	42, 97
	Hexanethiol	Ag QDs	2D superlattices	17
	Hexadecanethiol	Au sphere	Crystalline, Janus, and core–shell supraparticles	98, 105
		Ag octahedra		
			2D superlattices	
	DMAET	CdTe	2D free floating films	199
	p‐mercaptobenzoic acid	Ag complexes	f.c.c superlattices	76
	4‐mercaptobenzoic acid	CdTe	Thin film layers	221

### 3.1 Nanoparticle Surface Ligand Chemistry

Some molecular ligands, such as alkanethiols, have strong affinities to metal particle surfaces by forming covalent bonds. They can be introduced during nanoparticle synthesis. Upon purification, alkanethiols capped particles can self‐assembly to form high‐quality superlattice typically via drying‐mediated self‐assembly strategy.^60^, ^61^ However, in some instances the original protective ligands may be weak‐binding ligands (eg. CTAC, citrate) that are unsuitable for superlattice growth, and must be replaced by other surface binding soft ligands that are better suited for self‐assembly processes.

One can in principle do all the solid surface chemistry on tiny nanoparticle surfaces. One powerful way to chemically modify nanoparticle surfaces is by ligand exchange. The ligand exchange is based on the principle that stronger binding ligands can replace original weaker binding ligands forming “new soft shells”. Depending on functional moieties on the end of molecular ligands, various forces such as electrostatic,^62^ hydrogen bonding^63^ or covalent bonding^64^ have been used in ligand exchange. Among all these types of ligands involved in gold nanoparticles, thiolated ligands^57^ are the most popular choice in ligand exchange due to its highest affinity (approximately 200 kJ mol^−1^)^63^ to gold.

The ligand exchange can also occur in heterogeneous biphasic system.^63^, ^65^ One common example is the well‐known Brust method which involves usage of ammonium ion capping as a stabilizer/phase transfer agent followed by ligand exchange with thiolated ligands.^66^ The same concept holds for semiconductor quantum dots such as CdS, CdSe and CdTe, in which stronger binding molecules such as thiols can exchange with the original electrostatically‐stabilized TOP, TOPO or amines ligand shells.^67^, ^68^

### 3.2 Molecular Ligand‐to‐Ligand Interactions

**Figure 3:**
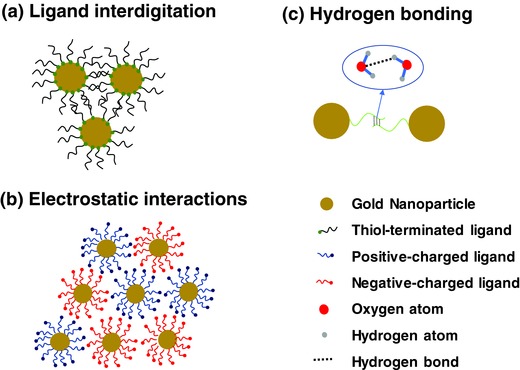
Molecular ligand‐ligand interactions towards nanoparticle self‐assembly. a) Balance between ligand‐ligand steric force and core‐core van der Waals forces lead to interdigitation of ligand in thermodynamic equilibrium. b) Electrostatic interactions between charged particles can lead to nanoparticle assembly. c) Directed assembly of gold nanoparticles due to the specific hydrogen bonding interactions.

The physical and chemical interactions between soft ligands play an important role in the formation of superlattices.^26^, ^69^, ^70^ The type of forces may include steric hindrance forces, electrostatic attraction/repulsion and hydrogen bonding. During the self‐assembly of nanoparticles, the core‐to‐core van der Waals (vdWs) forces, in particular for metallic nanoparticles, are usually very strong when particles are close enough to fall within vdWs well.^23^ Manipulation of the complex anisotropy of vdWs and other nanoparticle interaction forces has been proven to be a useful option to guide complex one‐dimensional assemblies.^71^, ^72^ An effective way to defy strong vdWs attraction is to introduce steric hindrance forces by using soft ligands (dominantly alkyl ligands). The ligand‐ligand steric forces can balance vdWs forces, leading to the ordered superlattice formation typically by a drying‐mediated self‐assembly process. The strong vdWs forces usually lead to the ligand interdigitation in the thermodynamically equilibrium (**Figure** **3**a).^26^, ^73^

An alternative way to manipulate nanoparticle self‐assembly is to introduce charges to ligand molecules. Different from van der Waals forces, electrostatic interactions can be attractive, repulsive or directional, and fine‐tuning electrostatics can enable high‐quality superlattice formation (Figure 3b).^23^ For example, diamond‐like lattice structures have been formed from charged, equally sized metal nanoparticles of two types (gold and silver). The formation of such non–close‐packed structures is a consequence of ligand‐mediated electrostatic interactions specific to the nanoscale, where the screening layer thickness is comparable to nanoparticle sizes.^74^

The specific inter‐ligand hydrogen binding can also be exploited to drive and direct the assemblies of nanoparticle superlattice (Figure 3c). While the non‐specific vdWs or electrostatic interactions represent the most common forces responsible for nanoparticle assembly, nanoparticles held by such forces are generally not fully controllable or stable on the macroscopic scale. In comparison, hydrogen bonding possesses specific molecular recognition capabilities, intermediate binding strength and reversibility, thus offering an efficient and flexible pathway for manipulation of superlattice assembly.^25^, ^75^, ^76^, ^77^, ^78^ A “squeezed” interparticle spatial model have been developed for a model system of alkanethiolate‐capped nanoparticles and carboxylic acid mediation agent, which concludes that the combination of both hydrogen bonding at the carboxylic acid terminal groups and cohesive vdWs forces from interdigitated alkyl chains were the responsible interparticle assembling forces that direct the formation of hexagonal close‐packed superlattices.^75^ In addition, the mediation of the hydrogen bonding network with hydrogen bonding agents allows one to structurally tailor the interparticle spacing within the superlattices. For example, fine tuning of the interparticle spacing can be achieved by monomolecular incorporation of various mediators between adjacent nanoparticles in the superlattices.^79^

In addition, some assemblies were prepared based on the interaction between stimuli‐responsive small molecular ligands.^25^, ^80^, ^81^ For example, metal nanoparticle supraspheres are assembled by the aggregation and cross‐link of photoactive transazobenzene dithiol (ADT).^81^ This light‐induced self‐assembly process was caused by rapid trans‐cis isomerization of ADTs molecules and dipole‐dipole interaction on the azobenzene units under UV irradiation.

### 3.3 Ligand Length Effect on Superlattice Assembly

The molecular chain lengths have been proven to greatly influence the structure and property of the assembled superlattice. Detailed studies have revealed that control over the interparticle spacing of the assembled superlattice over a limited range can be achieved by utilizing organic thiols with different chain length (C_6_ to C_20_) as a capping ligand.^82^, ^83^ For short ligands such as hexanethiol (C_6_), large attracting forces between nanoparticles resulted in aggregation or formation of small 3D superlattices. As the chain length increases (C_8_ to C_14_), the balanced interaction among particles led to self‐correction and organization, forming well‐ordered 2D hcp superlattices. It was demonstrated that the interparticle gap length (l) within the hcp superlattice arrays was linearly dependent on the number of carbon atoms per alkyl chain (n):^84^

(10) l = 0.83 + 0.122n

However, as alkyl chain length increases until more than 16 carbon atoms, it resulted in an abrupt increase in interparticle spacing without formation of any well‐ordered superlattices.^83^, ^84^ This chain length dependent phase transition phenomenon suggested that the long chain alkyl thiolates (>C_16_) exist in an extended, all‐trans‐ordered conformation, while chain disorders exists for short chain thiolates. This fact indicates that the interparticle interactions can be well‐controlled by using surfactants with different lengths, and finally leads to fabrication of tunable superlattice structures.

### 3.4 Ligand Number Density Effect on Superlattice Assembly

An optimum number density of soft ligand molecules is generally required for the successful growth of a well‐ordered superlattice.^73^, ^85^ For example, it has been demonstrated that the excess nonvolatile dodecanethiol ligand may provide a lasting wetting layer that giving nanoparticles a high surface mobility, thus allowing nanoparticles to diffuse into equilibrium site, forming highly ordered superlattice structure.^73^ In another study, the addition of excess oleic acid caused the tetragonal distortion, leading to the transformation of body‐centered tetragonal (bct) cuboctahedral PbS nanocrystals superlattice to a honeycomblike, bilayered hexagonal lattice.^86^ In addition, the evolution of Pt nanoparticle superlattices with increasing pressure was demonstrated by investigating pressure‐induced mechanical behavior of two superlattices with different content of oleic acid ligands. Modulation of ligand density in combination with pressure can tune the threshold for superlattice structure and mechanical properties changes, potentially leading to new functionalities.^87^ In some cases, surface binding ligands need to be partially stripped off to promote 1D assembly by enabling directional dipole‐dipole interactions.^71^, ^72^, ^88^ In general, the presence of surface capping ligands may contribute to strong electrostatic or steric repulsions that over‐counteract the electric dipole moments which are predominantly present in semiconductor nanoparticles. Partial removal of the capping ligand increased the magnitude of the dipole moment such that the energy for dipole‐dipole attraction is greater than that of thermal motion.^89^

### 3.5 Various Lattice Structures with Different Molecular Ligands

By precisely controlling the type and length of soft ligands, the lattice structure of nanoparticle superlattice can be manipulated. As the most commonly used ligands, alkylthiol (e.g., propanethiol, hexanethiol, decanethiol, dodecanethiol, and hexadecanethiol, etc.) has been used to rationally construct 2D hexagonal closely packed superlattices^17^, ^18^, ^35^, ^56^, ^58^, ^73^, ^90^, ^91^, ^92^ and 3D superlattices^55^, ^93^ with various lattice structures. With the right control over the superlattice assembly kinetics such as flux of particles into the interface (*f*) and interfacial diffusion length (δ), it is possible to form 2D freestanding superlattice membranes.^90^

In addition, molecular ligands have been used to guide the self‐assembly of multicomponent building blocks (with various size, morphology, or composition) in one system. This strategy has created quasicrystalline binary nanocrystal superlattices and ternary nanocrystal superlattices.^94^, ^95^, ^96^ During the crystallization, the electrical charges, together with entropic, van der Waals, steric and dipolar interactions, played roles in determining the final lattice structures,^94^, ^97^, ^98^ such as isostructural with NaCl, CuAu, AlB_2_, MgZn_2_, MgNi_2_, Cu_3_Au, Fe_4_C, CaCu_5_, CaB_6_, NaZn_13_, and *cub*‐AB_13_ compounds,^10^, ^42^, ^97^ and dodecagonal quasicrystalline superlattice et al.^94^

The soft ligands are able to alter the charges on constituent nanoparticles and direct the lattice structure. For example, reproducible switching between different lattice structures was observed by controlling the amount of ligands, such as carboxylic acids, tri‐*n*‐octylphosphine oxide (TOPO) or dodecylamine.^42^ The co‐assembly of negative charged PbSe and Pd nanoparticles lead to dominant MgZn_2_ and cuboctahedral AB_13_ lattices. However, the same nanoparticles preferred formation of orthorhombic AB‐ and AlB_2_‐type superlattices after capping with oleic acid, and assembled into NaZn_13_‐ or cuboctahedral AB_13_‐type lattices after the capping with dodecylamine or TOPO. In addition, by using oppositely charged ligands, non‐close‐packed sphalerite (diamond‐like) crystals can be formed through the nanoscale electrostatic effects. For example, positively charged TMA‐capped AgNPs (HS(CH_2_)_11_NMe_3_^+^Cl^−^) and negatively charged MUA‐capped AuNPs (HS(CH_2_)_10_COOH) co‐assembled into diamond lattice.^74^ In this lattice structure, each nanoparticle was surrounded by four oppositely charged nanoparticles at the vertices of a tetrahedron. This demonstrated that the capping ligand can effectively mediate the electrical charges of nanoparticles, and direct the superlattice self‐assembly.

### 3.6 Assembling Anisotropic Nanoparticles With Molecular Ligands

The soft ligands also guided the assembly of anisotropic nanorods and controlled the superlattice structure. By using the alkyl phosphonate as capping ligands, CdS NRs assembled into hexatic smectic‐*B* (hex*B*) liquidcrystalline phase. However, if the capping ligands were changed into metal chalcogenide complexes (e.g., didodecyldimethylammonium^+^‐AsS_3_^3−^), CdS NRs formed the smectic‐*I* (Sm*I*) phase,^99^ which can be treated as the tilted analogue of the hex*B* phase. The formation of tilted liquid‐crystalline phases facilitated the exposure of capping ligands to the solvent, which decreased both surface energy and steric constraint at the side surface of NRs. The ligands also affected the directional self‐assembly of gold nanorods (AuNRs) into highly ordered 2D/3D superlattices. Cetyltrimethylammonium bromide (CTAB) is the most common ligand for AuNRs that binding on the surface with a bilayer fashion, and acting as shape‐inducing agent during seeded growth synthesis.^100^ However, CTAB‐capped AuNRs would form disordered aggregates during drying‐mediated assembly. After ligand exchange of CTAB into cationic gemini surfactants (e.g., (oligooxa)alkanediyl‐α,ω‐bis(dimethyldodecylammonium bromide), 12‐EO_x_‐12), the 12‐EO_1_‐12‐capped AuNRs formed long‐range order vertically aligned arrays with a hexagonal close‐packed geometry.^101^ The alignment of the nanorods was critically dependent on the concentration and surface capping ligand of the AuNRs. By dropcasting AuNRs on different substrates, small side‐by‐side assemblies were formed at low concentration, whereas high concentration of AuNRs resulted in assembly of near perfect vertical alignment of 3D AuNRs superlattice with hexagonal close packed arrangement. Such highly ordered standing AuNRs structure was attributable to the van der Waals hydrophobic interactions between interpenetration of gemini surfactant alkyl chains, as well as electrostatic interactions at the NR surface level originated from two charged capping sites of the ligand.

In addition to NRs, other anisotropic building blocks such as PbS and Cs PbBr_3_ nanocubes, cubic and polyhedral CeO_2_ nanoparticles, iron oxide truncated nanocubes, Ag octahedra and Au rhombic dodecahedra, octahedra, cubes can be successfully assembled into 1D, 2D, and 3D superlattice using oleic acid, alkylthiols, and cetylpyridinium chloride as capping ligands.^102^, ^103^, ^104^, ^105^, ^106^ A chemical approach was utilized to tailor the surface hydrophobicity of Ag octahedral with increasing chain length of alkylthiols, leading to a continuous structural evolution of the wafer‐scale 2D plasmonic superlattices.^105^ In another study, the ligand solubility and effective mean size played important roles in determining the resulting superlattice structures of oleate‐covered cubic and polyhedral CeO_2_ nanoparticles.^103^ These results indicated the important role of molecular ligands in the structural ordering of nanoparticles.

## 4 Polymer‐Mediated Nanoparticle Superlattices

Using molecular ligands, one of its major limitations is the capability to tune the inter‐particle spacing over a large regime owing to their limited length. For example, in alkyl‐based fabrication of superlattices, the inter‐particle spacing is constrained to a small range (≈1.2 – ≈2.3 nm).^83^ In addition, it is often challenging to use short molecular ligands to direct large‐sized nanoparticles to form superlattices. These limitations could be overcome by using polymeric ligands because their lengths can be controlled to much longer regimes than molecular ligands. To date, various polymeric ligands have been used various conjugation methods to anchor various nanoparticle building blocks to form 1D, 2D and 3D superlattices.^107^, ^108^, ^109^, ^110^ Some representative examples are summarized in **Table** **2**.

**Table 2:** Summary of different types of polymers that have been used in polymer‐based nanoparticle superlattices

Polymer ligand	Nanoparticle type	Conjugation method	Nanoparticle assemblies	Reference
**One Dimensional Assemblies**				
Poly(vinyl pyrrolidone)	Gold nanosphere	Grafting to	1D strings	125
	Gold nanorods	Grafting to	1D tip‐to‐tip strings	125
		Templated	1D end to end strings	222
Polyethylene glycol‐thiol	Triangular prisms	Grafting to	1D Vertex to vertex superstructures	125
Polystyrene‐thiol	Gold nanorod	Grafted to nanorod ends	1D chains, rings, bundled chains	69, 127, 128, 204, 223
Poly(N‐isopropylacrylamide)‐thiol	Gold nanorod	Grafted to nanorod ends	1D end to end chains	134
Polystyrene‐block‐poly(acrylic acid)	Gold nanoparticles	encapsulation	Dimers and trimers	224
**Two Dimensional Assemblies**				
Poly(methyl methacrylate)	Gold nanospheres	Grafting from	2D arrays	111
Poly(methyl methacrylate)/Poly(ethylene glycol)‐thiol	Gold nanospheres	Grafting to & grafting from	2D responsive plasmonic arrays	113, 225
			2D hexagonal superlattice	
	Gold nanorods	Grafting to & grafting from	2D responsive plasmonic arrays	111
Polystyrene‐thiol	Gold nanospheres	Grafting to	2D superlattice sheets	49, 130, 190
	Gold nanorods	Grafting to	Horizontally and vertically aligned 2D sheets	12
	Gold nanobipyramids	Grafting to	2D sheets	131
	Gold nanocubes	Grafting to	1D plasmene nanoribbon, 2D plasmene sheet, 3D plasmene origami	20
Poly(oxypropylene)diamines	Gold nanospheres	Grafting to	Interparticle spacing controllable 2D monolayers	122
Poly(N‐isopropylacrylamide)‐thiol	Silver nanoparticles	Grafting to	Thin films of arrays with fcc cubic structure	209
(11‐mercaptoundecyl) tetra (ethylene glycol)	Gold nanospheres	Grafting to	Island‐like clusters	125
	Gold nanorods	Grafting to	Side by side aggregates	125
Poly(styrene‐b‐ethylene propylene)	Gold and silica nanospeheres	Templated	2D sheets of alternating gold and silica spheres	119

### 4.1 Surface Chemistry: Grafting of Polymer to Nanoparticle Surface

**Figure 4:**
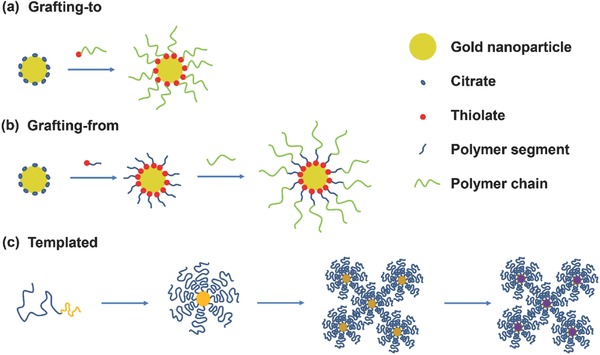
Schematic illustration of polymer grafting approaches used in superlattice community. a) Grafting‐from approach. b) Grafting‐to approach. c) Synthesis of nanoparticle from precursor within the assembled block copolymer matrix domain. The schematics are not drawn to scale and is modified. Adopted with permission.^28^, ^114^ Copyright 2011, MDPI. Copyright 2013, The Royal Society of Chemistry.

Similar to molecular ligands, grafting of polymeric ligands to nanoparticle surface to form densely capped soft corona shells is critical for superlattice formation. To date, various approaches such as grafting‐from^111^ and grafting‐to^108^ have been reported for homogenous and direct attachment of polymer chains to nanoparticles. The former relies on a polymerization reaction process to grow cross‐linked polymer shells from nanoparticle surface (**Figure** **4**a). However, the success rate of such grafting process to yield well dispersed polymer capped nanoparticles is limited due to the instability of initiator‐particle bond under high temperature.^112^ The latter grafting process relies on a versatile post synthesis ligand exchange processes to displace weakly chemisorbed surfactants on nanoparticle surface with thiolated polymer ligands, forming densely polymer‐capped nanoparticles (Figure 4b). Both of these grafting‐from and grafting‐to processes have been successfully demonstrated to be able to form monolayer superlattices on oil/water interface^113^ or millimeter‐scale 3D superlattice arrays by homogeneous deposition.^110^ By conjugating poly(ethylene glycol) and a polymerization initiator onto nanoparticle surface (grafting to), the surface initiated polymerization (grafting from) then leads to capping of AuNPs and AuNRs with amphiphilic binary polymer brushes.

### 4.2 Block‐Copolymer Templated Assemblies

Block copolymers are a unique class of linear copolymers in which two or more chemically distinct monomer units are grouped together to form blocks of repeating units along the polymer chain. One of the main advantages which had motivated various researches to utilize these interesting materials as soft templates is the ability to provide the organization of block copolymers over large areas.^114^ One of the earliest investigations on such system involves the direct integration of precursor reagents into preformed block copolymers lattices, thus providing compatibility during formation and entrapment of nanoparticles within the polymer matrix (Figure 4c).^115^, ^116^ For example, acid‐base interactions of gold chloroauric precursor with basic groups of copolymers such as polyvinylpyridines allow the preloading of precursor into the polymeric matrix. By proper optimization of precursor concentration and crucial kinetic parameters such as nucleation time and rate of precursor diffusion into the polymer microdomain, the ideal condition of a single nanoparticle growth within a microdomain could be achieved.^114^, ^117^

**Figure 5:**
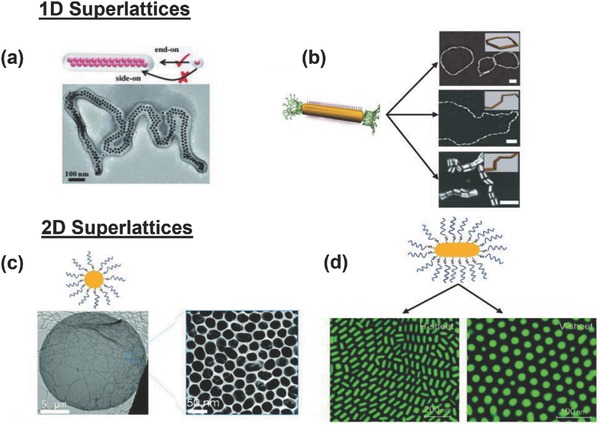
Polymer‐based 1D and 2D nanoparticle superlattices. a) 1D assembly of nanoparticle micelles into chains. Reproduced with permission.^120^ b) 1D assembly of PS end‐grafted nanorods into rings, strings and bundles. Reproduced with permission.^204^ Copyright 2007, Nature Publishing Group. c) TEM image of a 2D monolayer superlattice sheet self‐assembled from PS‐capped nanospheres. High magnification TEM image of part a, showing hexagonally packed lattice structure with uniform interparticle spaces. Reproduced with permission.^49^ Copyright 2011, American Chemical Society. d) SEM images of horizontally (left) and vertically (right) aligned PS‐capped nanorods. Nanorod assembly favors horizontal alignment at room temperature, whereas the thermodynamically stable vertical alignment is preferred at 60 °C. Reproduced with permission.^12^ Copyright 2011, American Chemical Society.

The more widely exploited strategy involves the incorporation of pre‐synthesized nanoparticles into block copolymers patterns.^114^, ^118^, ^119^ The localization of ex situ synthesized nanoparticles within the block copolymer domains requires significant considerations on the entropic and enthalpic interactions between nanoparticles and polymer blocks. For example, by using hydrophobic ligands such as polystyrene‐block‐poly(acrylic acid) (PSPAA) in a polar solvent, the polystyrene blocks aggregates into micelles due to van der Waals and hydrophobic interactions, with the size limited by the strong repulsion by the tethered poly(acrylic acid). With acid treatment, the transformation of PSPAA micelle from spheres to cylinders induced the selective linear assembly into 1D chains of nanoparticles (**Figure** **5**a).^120^

In addition, the combination of the self‐consistent field theory for block copolymers and density functional theory for nanoparticles has predicted that the location of the self‐assembled nanoparticles will be dependent on the particle size.^121^ A study conducted on nanoparticle‐diblock copolymer blends using a ternary system of polystyrene‐block‐poly(ethylene propylene) (PS‐b‐PEP), 3.5 nm gold nanoparticles and 21.5 nm silica nanoparticles demonstrated the influence of entropic contributions on the assembly process.^119^ The larger silica nanoparticles were found to predominantly accumulate to the centre of the PEP domain to minimize chain stretching that overcomes the loss of translational entropy of the particles. On the other hand, the smaller gold nanoparticles experienced less stretching and are driven by the translational entropy to assemble between PS‐PEP polymer domains.

### 4.3 Polymeric Ligand‐to‐Ligand Interactions

Polymeric ligand‐to‐ligand steric hindrance forces are often used to counteract strong vdWs attractions, enabling superlattice formation. By manipulating the polymeric chain length, the steric repulsion between close packed particles can be controlled, hence dictating the final lattice structure and particle orientation of the assembled superlattices. In one example, poly(oxypropylene)diamene ligands were used to direct gold nanospheres to self‐assembly into 2D and 3D structures, in which the interparticle spacing was observed to increase from ≈3 nm to ≈7 nm with increasing molecular weight from 230 g mol^−1^ to 4000 g mol^−1^.^122^

Both computational modeling and experimental studies demonstrated that the polymer ligand length dictates the final assembled nanostructure, in which it can be modulated into aggregates, short strings or sheets.^123^, ^124^ The dipole‐like interactions caused by rearrangement of polymer grafts at nanoparticle surface resulted in 1D chains of gold nanospheres within a poly(vinyl pyrrolidone) (PVP) matrix.^125^, ^126^ Subsequent change to a shorter PEGylated alkanethiol polymer grafts results in an increase in nanoparticle packing density, thus resulting in the nanospheres to form island‐like clusters. Similar experiments with gold nanorods and silver triangular nanoprisms obtained identical results,^125^ in which the polymer‐polymer steric repulsions of long polymer grafts leads to 1D string assemblies with tip to tip and vertex to vertex orientations, respectively. Replacement to short polymer grafts resulted in a nanoparticle orientation that favors side to side interactions due to the strong van der Waals attractions.

The hydrophobic/hydrophilic interactions between polymeric ligands can be used to use direct 1D superlattice formation. For example, the ends of gold nanorods (AuNR) could be site‐selectively functionalized with polystyrene, rendering the ends hydrophobic while the longitudinal sides remained capped by hydrophilic CTAB.^127^, ^128^ By regulating the conditions of the organic solvent containing AuNRs, nanoparticle self‐assembly was triggered by the selective hydrophobic and hydrophilic properties to obtain various orientational assemblies such as rings, chains and bundles (Figure 5b). The role of hydrophobic interactions in driving the self‐assembly of polystyrene ligand capped gold nanoparticles into gold clusters has also been shown in a THF/water system.^129^ The hydrophobic PS chains compress and attract to each other to expel solvent molecules, this minimizing the free energy of the system. The clusters are then stabilized via addition of a polymeric surfactant comprising of both hydrophobic (PS) and hydrophilic (poly(acrylic)) blocks, allowing the sequestration of clusters inside the hydrophobic core, while the outer hydrophilic surface ensures stability. The hydrophobic interactions can be modulated by controlling the length of the grafted polymer or the amount of water content, thus allowing one to tune the size and interparticle spacing of the assembled clusters.

### 4.4 Polystyrene Based Approach to Nanoparticle Superlattices

Among various polymeric ligands, thiolated polystyrene (SH‐PS) is an attractive choice of ligand to facilitate nanoparticle self‐assembly due to its several advantages. For instance, 1) it is commercially available with different molecular weights that allow precise tuning of ‘interaction softness’ for tailoring of interparticle spacing; 2) the thiol head‐group provides strong binding affinity of fold nanoparticle surfaces; and 3) SH‐PS is very soluble in organic medium but highly immiscible with water, thus facilitating interfacial self‐assembly. As a result, SH‐Ps can serve as a great model system for a high number of quality nanoparticle superlattices reported to date (see Table 2).

An efficient two‐step grafting‐to process has been reported to prepare PS‐capped gold nanoparticles for monolayer assemblies of 2D arrays.^12^, ^49^ Thorough studies revealed that the PS ligands exhibited brush like conformation irrespective of the ligand length, and treated as constrained polymer chain in a cylindrical‐to‐conical geometry.^130^ Using a drying mediated, entropy driven self‐assembly approach at the air‐water interface, it is possible to grow 2D free‐standing nanoparticle superlattice sheets.^12^, ^49^ This approach appears to be general, and both nanospheres and nanorods could be directed to form high‐quality superlattice sheets (Figure 5c and d). For the gold nanospheres of different sizes (13 & 26 nm), it was found that the polystyrene ligand experienced different degrees of compression: smaller interparticle spacing was obtained for larger nanospheres as they experienced a larger degree of compression due to strong core‐to‐core vdWs force. This simple yet robust polystyrene‐based entropy driven approach could be extended to anisotropic building blocks such as gold nanorods and gold nanobipyramids. Different from the site‐selective grafted nanorods in 1D assemblies, the nanorods/nanobipyramids utilized here were fully grafted with PS ligands via a ligand exchange process. Two distinct packing orders for gold nanorods – vertically and horizontally aligned superlattice sheets were obtained (Figure 5d). These two packing orders gave rise to distinct plasmonic properties: vertically‐aligned nanorod sheets exhibited only transverse resonance peak; whereas, horizontally‐aligned nanorod sheets exhibited both longitudinal and transverse modes.^12^ In the case of gold nanobipyramids, four packing orders including horizontal, circular, slanted, and vertical alignments were observed, leading to structure dependent SERS enhancements from different kinds of collective plasmonic coupling strength and modes.^131^

Recently, our research group introduced the ‘plasmene’ concept, in which a combination of bottom up PS‐based self‐assembly and top‐down lithography approach was utilized to fabricate 2D giant plasmene nanosheets, 1D plasmene nanoribbons and 3D plasmene origami.^20^ The giant 2D sheets comprised of nanocube particles assembled in an ordered packing were demonstrated to possess a single‐particle thickness with an aspect ratio of ≈75,000. Theoretical and experimental studies revealed that the sheets are able to support both localized gap plasmons and surface propagating plasmons, thus enabling their use as Surface‐enhanced Raman scattering (SERS) adhesives, and fiber‐plasmene coupler waveguide.

**Figure 6:**
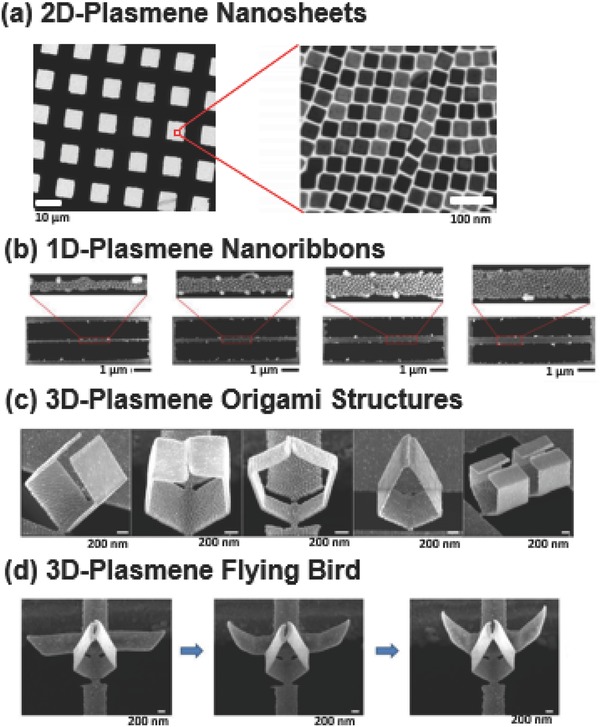
Polystyrene‐based plasmene superlattices. TEM images showing (a) free standing 2D plasmene nanosheets as fabricated on holey square copper grids. Magnified image shows the nanocube building blocks in highly ordered packing. These free‐standing nanosheets are then used for top‐down focused ion‐beam (FIB) patterning into (b) 1D plasmene nanoribbons with increasing widths, (c) 3D plasmene origami structures–cube, pentagon, hexagon, diamond and heart, and (d) 3D plasmene bird, showing the f lapping motion of the wings. Reproduced with permission.^20^ Copyright 2014, American Chemical Society.

Taking advantage of the mechanically robust, flexible and free‐standing nature of the 2D sheets (**Figure** **6**a), they can be further shaped with focused ion beam (FIB) lithography without fracture into 1D plasmene nanoribbons (Figure 6b) that exhibited graphene‐like width‐dependent plasmonics. Uniquely, the nanoscale interaction forces can be manipulated by inducing a local stress building via partial etching of the surface‐binding polystyrene ligands which resulted in controlled folding of the nanoribbons at various angles. This facilitated the engineering of different 3D plasmonic origami structures such as cube, hexagon, pentagon, heart and even a ‘flying bird’ (Figure 6c and d). Detailed theoretical studies revealed the folding‐induced plasmonic evolution, and we envision these foldable 3D structures will lead to a wide range of technological applications such as plasmonic sensing, communication and waveguide etc.

### 4.5 Stimuli‐Responsive Polymeric Ligands

Polymeric materials generally exhibit exceptional mechanical properties, which makes them an ideal choice to withstand various external stimuli such as high temperature or swelling that help to facilitate superlattice assemblies.^132^ One of the more common approaches is to graft thermal responsive polymers such as Poly(N‐isopropylacrylamide) (PNIPAM) to nanoparticle surface. PNIPAM is a kind of thermoresponsive polymer which possess a coil‐globule transition at its lower critical solution temperature. By homogenously coating a uniform layer of PNIPAM shell around nanoparticles, this shell can be utilized as a flexible spacer for nanoparticle assemblies.^133^ Ordered 2D nanoparticle monolayers can then be prepared by either spin coating or convective assembly before the removal of the PNIPAM shell via heat treatment. The lattice spacings of the assembled 2D nanoparticle arrays can be easily tuned by controlling the thickness of the PNIPAM coating. Alternatively, PNIPAM polymer can also be selectively grafted onto nanorod ends, thus allowing reversible assembly of nanoparticles via temperature control. Under NIR illumination, nanorods absorb and convert the irradiation into thermal energy, thus causing the hydrophilic to hydrophobic transition of PNIPAM.^134^ Hydrophobic‐hydrophobic interactions of the shrunken PNIPAM then guided the nanorod into end‐to‐end chain assemblies. The chain assembly can be easily reversed by the swelling of PNIPAM temperature is cooled to room temperature.

## 5 DNA‐Mediated Nanoparticle Superlattices

Attaching biomolecules such as DNA,^11^, ^46^, ^47^, ^135^ proteins^136^, ^137^, ^138^, ^139^, ^140^ and antibodies^141^, ^142^ to nanoparticle surfaces offer a unique specific route to control their assembly. Such assemblies are attractive because they can be programmed into complex structures, such as chiral architectures.^141^ Among various biological ligands, DNA is by far the most successful molecule in directing superlattice formation due to its unique linear structure and well‐controlled Watson‐Crick base‐pair forces.

DNA is a unique molecule which can have a molecular length even greater than that for typical polymers. The specific Watson‐Crick base‐pairing forces between DNA bases adenine (A), thymine (T), cytosine (C) and guanine (G) enables DNA to become a programmable molecule. These fascinating properties are being used by scientists for building novel designer materials at the nanoscale^143^ such as well‐defined nanoscale origamis^144^, ^145^, ^146^ and nanoparticle superlattices.^2^, ^7^, ^44^, ^46^, ^145^ The key to use DNA for self‐assembly processes lies with its molecular recognition programmability due to the specific Watson‐Crick pairing, namely the specific hydrogen bond binding between A and T, and C to G. Under optimum conditions, two complementary single stranded DNA will join together into a double helical structure. This programming capability have been harnessed to build unique superlattice structures including lattices that are not existing in nature.^21^

### 5.1 DNA‐Based Superlattice‐Fabrication Strategies

**Figure 7:**
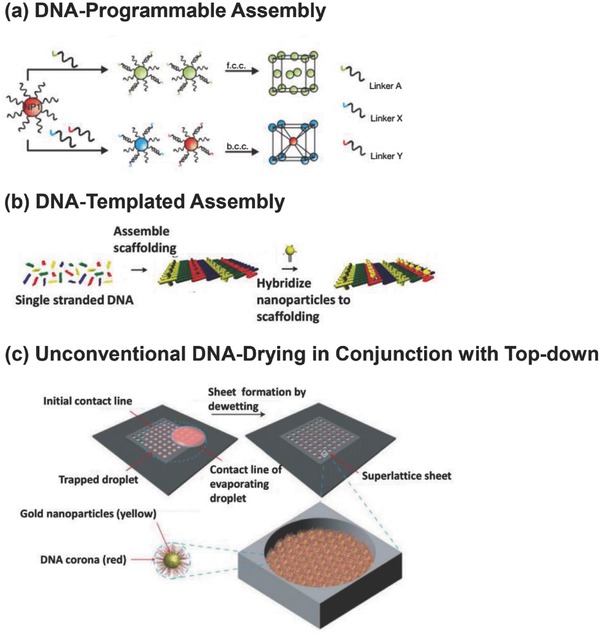
Schematic illustration of the 3 major routes to DNA‐mediated superlattices. a) Formation of nanostructures with different crystallographic lattices by programming DNA sequence. Reproduced with permission.^45^ Copyright 2008, Nature Publishing Group. b) Usage of multiple single‐stranded DNA into well‐defined scaffolds for nanoparticle assembly. Reproduced with permission.^154^ Copyright 2004, American Chemical Society. c) Evaporation mediated assembly of DNA‐capped gold nanoparticles into free‐standing DNA superlattices. Reproduced with permission.^11^ Copyright 2009, Nature Publishing Group.

There are three major routes in which DNA can be utilized for assembling nanoparticle superlattices, namely programming (**Figure** **7**a), templating (Figure 7b) and drying mediated assembly (Figure 7c). DNA has been known to possess unique molecular recognition capability as well as structural versatility which can be exploited to program and guide nanoparticles into ordered arrays. These unique features can be used to sequentially design and facilitate the various interactions and interplay between forces that are present within a DNA‐nanoparticle system. Mirkin et al.^47^ and Alivisatos et al.^46^ demonstrated organization powers of DNA molecules in programming materials synthesis in the mid‐1990s. The former reported on using DNA as a particle directing motifs to assemble nanoparticles into amorphous aggregates which are reversible at the DNA denaturation temperature, while the latter showed the nanoparticle linear self‐assembly into dimers and trimers.

In terms of conventional hybridization‐based approach, the wet nanoparticle superlattice crystals were demonstrated in 2008.^44^, ^45^ These breakthroughs stemmed from accurate temperature control over DNA hybridization. Synchrotron‐based Small‐angle X‐ray scattering (SAXS) studies revealed that temperature programming plays a pivotal role in forming ordered assemblies. The crystallization of DNA nanoparticles generally involves the initial formation of a disordered aggregate, followed by transformation into an ordered superlattice upon thermal annealing below its melting temperature. The temperature programming triggered a series of follow‐up development of other types of superlattices.^21^, ^147^, ^148^, ^149^, ^150^

An alternative way to manipulate nanoparticle ordered assembly is to use rigid DNA templates such as double (DX)‐ and triple(TX)‐crossover motifs and origami.^146^, ^151^, ^152^, ^153^, ^154^, ^155^ These rigid tiles enabled construction of ordered nanoparticle arrays with precisely defined inter‐particle spacing.^154^ Other complex assemblies such as 1D spiral chains,^156^ 2D rhombic lattice^157^ and 3D tubular assemblies^156^ of gold have been constructed with DNA tile‐templated self‐assembly. DNA origami is another versatile type of templates for assembly of nanoparticles.^144^, ^158^ While the conventional crossover tile strategy requires a two‐step process of tile construction followed by nanoparticle assembly, DNA origami utilizes a simple and versatile ‘one‐pot’ process to induce the folding of long genomic DNA scaffolds into desirable nanopatterns via short staple DNA strands. Multiple clusters, Ag‐Au heterodimers, heterotrimers and even complex structures such as helical plasmonic assemblies, hexagonal 2D lattices and tubes have been obtained from origami based assembly.^20^, ^159^, ^160^, ^161^ While DNA template engineering has shown great advantages in providing a rigid and versatile framework to spatially control nanoparticle assemblies, these strategies are generally limited to small‐scale superlattice assemblies due to size restriction by DNA scaffold.^45^

**Figure 8:**
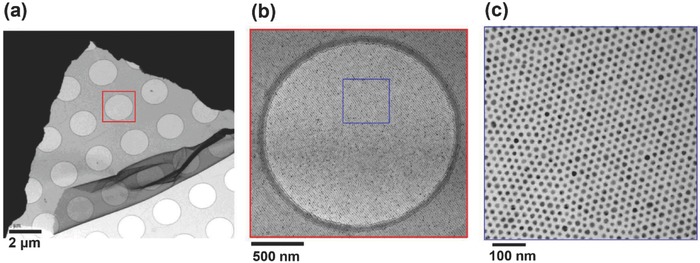
Transmission electron microscope images of giant DNA‐nanoparticle superlattice nanosheets under different magnifications. The giant nanosheets were obtained by simply dipping holey carbon substrate into a suspension of desalted DNA‐capped nanoparticles followed by drying in the air. Nanoparticle diameter is 12 nm; the ligand is thiolated 5′‐ATGGCAACTATACGCGCTAGAGTCGTTT‐3′.

The programming and templating routes exclusively take advantage of DNA hybridization forces for manipulation of nanoparticles, which provides the advantage of specificity. The disadvantage is that the assembling process requires aqueous buffered environments or the final products collapsed after drying, which limits their applications in solid state devices. Using an unconventional approach,^162^ DNA was utilized as a ‘dry ligand’ to entropically drive nanoparticles into highly ordered superlattices without the need of specific Watson‐Crick base pairing.^163^ This unconventional DNA‐based strategy could lead to giant superlattice nanosheets with a thickness below 20 nm but with a lateral dimension of millimeter scale (**Figure** **8**). More importantly, these DNA‐based nanoparticles superlattices could be shaped into nanoscale features^163^ or ultrathin nanosheets^11^ by combining with top‐down lithography. For example, top‐down fabricated micromoulds regulated local superlattice nucleation and growth events, enabling the formation of single‐particle‐width corrals, single‐particle‐thickness microdiscs and submicrometer sized supra crystals. When combined with top‐down fabricated microholes, it is possible to grow DNA‐nanoparticle superlattice sheets with ultimate thickness limit yet exhibiting strong mechanical strength and strong plasmonic coupling properties. These patternable and large‐area nanosheet structures and their associated properties couldn't be easily achieved using conventional DNA‐based strategies.

### 5.2 Various Lattice Structures Obtained from DNA‐Based Strategies

**Figure 9:**
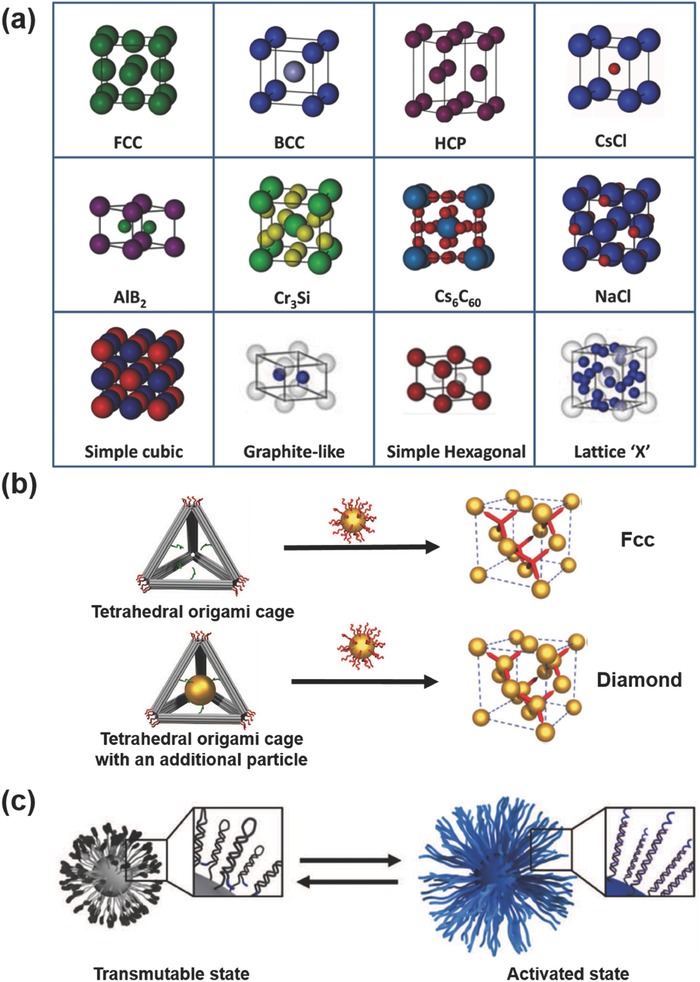
a) Different crystal lattice structures obtained using DNA ligands. By varying the DNA length, sequence, nanoparticle size and number of cores, a variety of different crystal structures can be obtained. Reproduced with permission.^7^ Copyright 2011, Nature Publishing Group. b) Assembly of nanoparticles on vertex of the tetrahedral origami cage with/without an additional nanoparticle, leading to the formation of f.c.c and diamond lattices. Reproduced with permission.^164^ Copyright 2016, The American Association for the Advancement of Science. c) Transmutable nanoparticles and their activation pathways. Reproduced with permission.^168^ Copyright 2016, The American Association for the Advancement of Science.

With DNA motifs, it is possible to obtain various lattice structures (**Figure** **9**a), including some interesting lattice “X” structures that are not existing in nature.^21^ In a single DNA linker assembly system, the equivalent binding affinities between nanoparticles led to formation of a close‐packed face‐centred‐cubic (f.c.c) structure.^45^ Alternatively, nanoparticle assembly through binary DNA linker strands favours hybridization that results in a non‐close‐packed body‐centred‐cubic (b.c.c.) structure. With another approach,^44^ the direct mixing of two different ssDNA‐capped nanoparticles, both with spacer regions of varying length followed by complementary base‐pairing sequences will bridge the nanoparticles together into a 3D b.c.c structured superlattices. A thermodynamic Wulff polyhedral structure with specific and uniform crystal habit have been achieved via slow cooling of complementary DNA functionalized gold nanoparticles.^147^ Different from the traditional annealing method which produces polycrystalline superlattices without any definite shape, both experimental and molecular dynamic simulation studies revealed that this slow‐cooling process enables a DNA driven assembly and crystallization which favors the most thermodynamically stable crystal structure. A rhombic dodecahedral nanostructure exhibiting a b.c.c lattice packing was obtained, whereas a CsCl lattice symmetry was observed when a binary system of 15 and 20 nm gold nanoparticles were used. A diamond family of lattices were assembled by using anisotropic tetrahedral origami cages as topological linkers between isotropic spherical gold nanoparticles.^164^ Assembling nanoparticles on each vertex of the tetrahedral cage resulted in the formation of f.c.c. lattice, while a diamond lattice was obtained by caging an additional nanoparticle inside DNA tetrahedron (Figure 9b). By replacing the basis particle or the guest particle with a smaller particle, two variant diamond lattices, namely a zinc blende lattice and a “wandering” zinc blende lattice were fabricated. Moreover, different crystallographic lattices can be assembled by employing different frame geometries, such as octahedron, cube, elongated square bipyramid, prism and triangular bipyramid.^165^ These results demonstrated the supremacy of DNA to regulate the recognition properties, surface energy of individual nanoparticles as well as the surface energy of the macroscopic nanoparticle assembly so that a specific structure can be achieved.

Since DNA is able to process a set of conformational changes in response to specific chemical stimuli, the controlling structure transformation of DNA based nanoparticle superlattices into multiple phases can be accomplished through shifting interparticle interactions. For instance, by insertion of a third nanoparticle component into binary superlattice at predefined positions, a reversible topotactic intercalation was achieved and five distinct ternary crystals were synthesized using this methodology.^166^ The crystalline structures can also be switched on demand via inputting specific types of DNA strand. The lattice transform from the original “mother” (CsCl) phase into various “daughter” (CuAu, hexagonal close‐packed, quasi‐2D, face‐centred cubic, and a cluster morphology) states, by selectively increasing particle‐particle attraction or/and repulsion.^167^ Recently, this conformational change of DNA capped nanoparticles was further extended to create crystal lattices from a nascent set of “transmutable “nanoparticles (Figure 9c). The interparticle bonding of DNA hairpin ligands anchored on nanoparticles can be selectively activated and deactivated in response to appropriate chemical cues. As a result, different types of superlattices were constructed tuning four factors: (1) the type of sticky ends that defines the type of bonding; (2) the effective stoichiometry of PAEs; (3) the density of bonding elements on individual particles, and (4) the hydrodynamic size of particles.^168^ These transformable assemblies of DNA capped nanoparticles with controllable kinetics indicate the versatility of DNA assembly strategy.

A set of critical design rules, as discussed earlier, were established as a guide for superlattice fabrication using DNA as a programmable linker, allowing one to tailor and dictate the crystallographic symmetry and lattice parameters.^7^, ^53^ The rules outlined a strategy to independently adjust the relevant crystallographic parameters such as particle size, periodicity and interparticle distance to obtain superlattice architectures with a tailorable and predictable fashion. New cubic lattice configurations which are generally not possible in nature have been engineered by utilizing hollow DNA nanostructures as a 3D spacer to selectively program voids into unique lattice structures (Figure 9).^21^ In addition to the sequence and structural tuning of DNA ligands, the crystallization of DNA‐nanoparticles into superlattices is also strongly influenced by the nanoparticle building block shape. By investigating the influence of anisotropic structures such as gold nanoprisms, nanorods, rhombic dodecahedra and octahedral on particle crystallization parameters, it was revealed that particle shape strongly affects the superlattice dimensionality, crystallographic symmetry and phase behavior.^148^ A strategy towards multifunctional and field responsive heterogeneous (magnetic, catalytic, fluorescent, plasmonic) nanoparticle superlattice via DNA and carboxylic based conjugation has also been reported.^149^ By using DNA as a programmable ligand, the properties of a mesoscale superlattices can be either plasmonic or photonic by controlling the spacing between the building blocks and crystal habit.^169^ For a binary A‐B nanoparticle superlattice, the number of grafted DNA per nanoparticle, f, is a crucial parameter to the outcome of the assembly behavior, ranging from finite sized clusters (f_A_ + f_B_ = 30) to weakly‐ordered (30 < f_A_ + f_B_ < 70–80) and well‐ordered superlattices (f_A_ + f_B_ > 70–80). For nanoparticle systems of different f_A_ and f_B_, short ssDNA motifs generally results in superlattices with compositional order, whereas long ssDNA motifs promotes long‐range lattice ordering.

### 5.3 Synchrotron Based Study of DNA Crystallization Dynamics

Synchrotron‐based small‐angle X‐ray scattering (SAXS) is a critical technique to reveal how DNA manipulate nanoparticle assembly in aqueous buffered environments^44^, ^45^ as well as in dehydrated state.^48^ High flux of X‐ray from Synchrotron enables acquisition of crystalline information almost instantaneously – a unique capability for the real time monitoring crystallization events.^48^ The real‐time probing of the entire drying process of DNA‐capped nanoparticles revealed the DNA‐mediated nanoparticle crystals to be soft with continuously scalable lattice constants with gradual transition from ‘wet crystals’ to ‘dry crystals’. Interestingly, comparison of Poly(dT) and palindromic DNA ligands showed both ligands could be used to produce soft superlattices but crystallization times were different.^48^ Synchrotron‐based SAXS is also critical for probing temperature‐induced crystallization events.^44^, ^45^ It is virtually impossible to probe complex temperature‐dependent nanoparticle crystallization in aqueous buffered environments with other techniques. Synchrotron‐based SAXS could provide important information on lattice structures, lattice constants, crystal sizes, etc.,^44^, ^45^, ^148^ which helps formulation of DNA‐based design rules.^53^

In addition to transmission SAXS in a droplet‐based^48^ or capillary tube‐based^53^ setup, synchrotron based Grazing‐Incidence Small‐Angle X‐ray scattering (GISAXS) could be used to probe the spatial crystallization events of DNA‐capped nanoparticles at air‐water interface of a sessile droplet.^170^ A raster scanning of the entire droplet allowed for revealing that crystalline Gibbs monolayers of DNA‐capped nanoparticle formed at the air/water interface. The formation of Gibbs superlattices was critically dependent on the ionic strength. The interparticle spacing of the Gibbs superlattice monolayers were showed to be programmable by regulating by both ionic strength and DNA sequence length. These previous studies show that both temporal and spatial crystallization information can be obtained by Synchrotron‐based X‐ray techniques.^48^, ^170^

## 6 Ligand Length on Superlattice Properties

The assembly of nanoparticles into ordered superlattices have led to the discovery of novel and remarkable collective properties that are different to that of individual nanoparticles or corresponding bulk materials. A number of parameters^71^, ^171^, ^172^ can affect superlattice properties, including particle crystallinity,^173^ lattice structures^174^, ^175^ and interparticle spacing. This can be directly related to the length of the soft capping ligands, which plays a critical role in resulting superlattice properties by controlling interparticle spacing. In ensembles of magnetic nanoparticles, the capping ligand influences the packing density, packing order, and lattice structure of the assemblies, leading to interesting magnetic properties such as increase in blocking temperature or change in shape of hysteresis loop.^176^, ^177^, ^178^, ^179^, ^180^, ^181^ For instance, in a system involving FePt nanoparticle superlattices, the exchange of ligand from oleic acid and oleyl amine to hexanoic acid or hexylamine led to a change in interparticle spacing from ≈4 to ≈1 nm, and a transition change in superlattice structure from hexagonal to cubic packing.^182^ Further thermal annealing led to the internal structure conversion from a disorded face‐centered‐cubic phase to ordered face‐centered tetragonal phase, and renders the superlattice with ferromagnetic properties.

The surface‐binding soft ligands of quantum dots (QD) dictate the properties of QD superlattices in many ways, since the ligands determine their solubility in a particular medium, as well as mediate the energy and charge transfer with surrounding environment. Short molecular ligands such as thioglycolic acid, sodium citrate^183^ and hydrazine molecules^184^ often lead to superlattices with small interparticle distance, hence, enable strong interparticle coupling interactions. For example, the charge transport properties in a nanoparticle superlattice are highly dependent on interparticle spacing.^185^ The use of short ligands could improve charge carrier mobility,^184^, ^186^ enabling the possibility of electronic tunneling through the short ligand layer in a superlattice system.^187^ Another interesting propery of QD superlattices is the fluorescence resonance energy transfer from an excited state donor to a ground state accceptor.^188^, ^189^ The short interparticle distances were found to be responsible for improving the energy transfer efficiency in QD assemblies, in particular for chain structures, due to the strong coupling of the donor and acceptor excited states.

The use of long capping ligands such as polymer and DNA often leads to highly‐ordered superlattices but with weak interparticle coupling interactions. The charge carrier transport across polymer‐ or DNA‐based superlattices has yet to be reported, possibly due to the large interparticle spacing. Nevertheless, the spacing is still within strong plasmonic coupling range.^11^, ^12^, ^49^ Tunable plasmonic properties of both polymer‐ and DNA‐based superlattices have been demonstrated previously. Another advantage of long ligands is that they can maintain overall superlattice integrity and mechanical compliance by strong ligand‐ligand interactions.^11^, ^12^, ^49^ For instance, polymer length was proved to play a role in determining mechanical properties of nanoparticle superlattices.^190^

## 7 Future Perspectives

The ability of assembling nanoparticles to form high‐quality superlattices opens up a new route in designing novel materials enabling applications that are impossible or difficult to achieve with conventional materials. However, the tiny nanoparticles are extremely difficult to manipulate due to complex nanoscale forces occurring at different temporal and spatial scales. To date, how to assemble nanoparticles into well‐defined structures remains an unresolved issue. Over the past two decades, a number of physical approaches using magnetic field, electrical field, or fluidic dragging forces, etc., have been attempted, however, the success is rather limited. In contrast, soft ligand‐based chemical approaches appear to be more promising, as seen from many recent exciting results reviewed in this paper.

Ligands such as linear molecules, polymers and DNA are virtually nanoscale soft building blocks; on the other hand, quantum dots, metallic nanoparticles, magnetic nanoparticles are nanoscale hard building blocks. The complex interactions among these hard building blocks could be controlled to a certain degree by using soft ligands. Soft ligand‐to‐ligand interactions including steric hindrance, hydrogen bonding, electrostatic attraction/repulsion and bio‐recognition forces such as Watson‐crick base‐pairing forces have been successfully harnessed to balance strong core‐to‐core vdWs forces or dipolar forces. This enables the assembly of nanoparticles into well‐defined 1D, 2D and 3D superlattice structures. In some cases, unprecedented lattice structures could be obtained.^21^ Some of these superlattices are robust enough, allowing for investigating unusual mechanical, plasmonic, conducting properties from superlattice assemblies.^3^, ^5^, ^11^, ^12^

Despite these stimulating advances, plenty of challenges need to be overcome before translating these superlattice structures into real‐world applications. Virtually, there are no single approaches enabling multiscale control over superlattice growth. Drying‐mediated approaches can sometimes lead to large‐area superlattices but only lead to limited lattice structures (fcc, hcp or bcc); The applications of molecule‐mediated nanoparticle superlattices are generally restricted due to the constrained range of interparticle spacing limited by the molecular chain length; Polymer‐mediated or DNA‐programmed approach allows a better range of control over the interparticle spacing, but the resulting interparticle coupling properties of the superlattices will be weak due to the increased spacing; combined top‐down and bottom‐up approaches enabled the control over superlattice growth at the nano‐, micro‐scale, forming some patterned superlattice structures, however, defects and yields remain the bottlenecks; some heterogeneous superlattice systems have been obtained with both molecular and DNA ligands, however, current success is limited to a few building blocks lack of generality.

It is currently highly demanding to develop multiscale experimental techniques and multiscale modeling approaches to understand soft‐ligand‐based self‐assembly of nanoparticle superlattices. To date, soft ligand‐based approaches have enabled nanoscale control over nanoparticle assemblies to a large degree. Further work will need to figure out ways to direct ‘site‐specific’ crystallization to position superlattices at desired locations in a defect‐free manner. This can be achieved by performing an in‐depth real time in situ study of individual nanoparticles to directly monitor the trajectory of nanoparticle motions. The recent development of liquid phase transmission electron microscopy has made it possible to provide quantitative insights to the interactions involving soft ligands, nanoparticles, as well as the structuring kinetic parameters that govern the evolution of a nanoparticle system from a disordered to ordered state.^191^, ^192^, ^193^, ^194^, ^195^, ^196^ However, these recent studies have been limited to systems involving single composition nanoparticles with simplistic shapes such as gold/platinum nanospheres, gold nanorods and CdSe/CdS octapods. These particle shapes remain only a small fraction of the nanoparticle library, hence future work may extend to exploration of diverse particle geometries, material and soft ligand composition, as well as hybrid binary or tertiary systems. Deeper understanding of nanoparticle self‐assembly may also be obtained via other experimental techniques such as SAXS, 3D structural tomography, optical microscopy as well as combining with multiscale modeling and simulation using molecular dynamics, coarse‐grain model in conjunction with Monte Carlo technique to reveal important information on molecular, nanoscale, mesoscale to macroscale assembly of nanoparticle superlattices. This may in turn lead to discovery of new design rules to guide experimental design for realization of multiscale control over self‐assembly of nanoparticles.

## Conflict of Interest

The authors declare no conflict of interest.
